# HPV infection and p53 and p16 expression in esophageal cancer: are they prognostic factors?

**DOI:** 10.1186/s13027-017-0163-4

**Published:** 2017-10-13

**Authors:** Allini Mafra da Costa, José Humberto Tavares Guerreiro Fregnani, Paula Roberta Aguiar Pastrez, Vânia Sammartino Mariano, Estela Maria Silva, Cristovam Scapulatempo Neto, Denise Peixoto Guimarães, Luisa Lina Villa, Laura Sichero, Kari Juhani Syrjanen, Adhemar Longatto-Filho

**Affiliations:** 1 Teaching and Research Institute, Barretos Cancer Hospital – Pius XII Foundation, Rua Antenor Duarte Vilela, 1331, Dr. Paulo Prata, Barretos, São Paulo 14784-400 Brazil; 2 Cancer Registry, Barretos Cancer Hospital – Pius XII Foundation, São Paulo, Brazil; 3 Molecular Oncology Research Center, Barretos Cancer Hospital – Pius XII Foundation, São Paulo, Brazil; 4 Department of Endoscopy, Barretos Cancer Hospital – Pious XII Foundation, Barretos, São Paulo, Brazil; 5 Molecular Biology Laboratory, Center for Translational Research in Oncology, Instituto do Câncer do Estado de São Paulo – ICESP, São Paulo, Brazil; 6 Department of Radiology and Oncology, School of Medicine, University of São Paulo, São Paulo, Brazil; 7 Department of Clinical Research - Biohit Oyj, Helsinki, Finland; 8 Medical Laboratory of Medical Investigation (LIM) 14, Department of Pathology, Faculty of Medicine, University of São Paulo, São Paulo, Brazil; 9 Research Institute of Life and Health Sciences (ICVS), University of Minho, Braga, Portugal; 10 ICVS / 3B’s - Associated Laboratory to the Government of Portugal, Braga/Guimarães, Portugal

**Keywords:** Human Papillomavirus, Esophageal cancer, Survival

## Abstract

**Background:**

Esophageal squamous cell carcinoma (ESCC) is a highly lethal malignant tumor. Currently, Human papillomavirus (HPV) is suggested as a potential risk factor for esophageal cancer (EC) in addition to the classic risk factors, alcohol and tobacco, but this hypothesis still remains contradictory. We sought to investigate wether HPV and well-known biomarkers (p16 and p53) and patient-related factors that may have impact on survival of ESCC.

**Methods:**

We conducted a prospective cohort study. By using multiplex PCR, we determined the prevalence of high risk HPV in ESCC, and evaluated the immunohistochemical expression of p16 and p53, molecular markers related to esophageal carcinogenesis in order to verify the potential influence of these variables in patients’s survival. Survival rates were estimated using Kaplan-Meier methods. A multivariate confirmatory model was performed using Cox proportional hazards regression.

**Results:**

Twelve (13.8%) of 87 patients were HPV-DNA positive. Positive reactions of p16 and p53 were 10.7% and 68.6%, respectively. Kaplan-Meier analysis indicated that men (*p* = 0.025) had poor specific-cancer survival and a shorter progression-free survival (*p* = 0.050) as compared to women; III or IV clinical stage (*p* < 0.019) had poor specific-cancer survival and a shorter progression-free survival (*p* < 0.001) compared to I and II clinical stage; not submitted to surgery (<0.001) and not submitted to chemoradiotherapy (*p* = 0.039) had a poor specific-cancer survival, as well. The multivariate analysis showed that HPV, p16 and p53 status are not predictive parameters of progression-free and specific-cancer survival.

**Conclusion:**

HPV infection and p53 and p16 expression are not prognostic factors in ESCC.

## Background

Presently, esophageal cancer (EC) is regarded as an important public health problem worldwide, being considered the eighth most common type of cancer and the sixth leading cause of cancer death according to estimates by GLOBOCAN 2012 [1].

Despite recent advances in multidisciplinary treatments, including radical surgical resection, chemotherapy and radiotherapy, the 5-year survival rate of patients with esophageal squamous cell carcinoma (ESCC) remains being less than 30%, and this is due mainly to atypical early symptoms, middle-to-late stage diagnosis, low treatment remission rates and high local recurrence rates, requiring the identification of a suitable biomarker to predict their long-term survival [2, 3].

Recently, evidence suggests that human papillomavirus (HPV) may play an important role in ESCC development; a number of studies in this area has increased steadily, as evidenced in several reviews [4–9]. First descriptions of oral lesions associated with HPV were preceded by reports that suggested the involvement of viruses in the development of benign [10] and malignant [11] lesions of the squamous epithelium of the esophagus. These initial observations were based on the report of morphological similarities between HPV lesions in the genital tract (warts) and esophageal papillomas [10, 11].

The first report that demonstrated the presence of HPV in ESCC occurred more than 30 years [10]; however, its prevalence is significantly variable among different geographical regions, and its role in carcinogenesis is still a matter of debate. Although the number of studies and interest in the subject has increased in recent years, literature is still controversial [12]. Data accumulated reflects a trend linking HPV infection and EC in high risk areas, whereas in low-risk areas such association was not evident [13].

The molecular genetic background of ESCC, mainly researches on protein alterations, has been widely studied and may assist in the prognosis of patients [14]. Proteins such as p53, p16 and others have been considered as prognostic factors for ESCC [15].

The differential expression of the tumor suppressor protein p53 is one of the commonest abnormality in several cancer types, including EC, and its mutation is mainly related to cell invasion and metastasis, as well as being related to advanced stages of the disease [14]. These mutations can lead to an increase in expression of p53, which accumulates in the nuclei and can be detected by immunohistochemistry (IHC) methods [16, 17]. The p16 protein expression is frequently used as a surrogate marker for HPV infection, and was shown as a marker for responder and better prognosis among head and neck squamous cell carcinoma patients who underwent radiotherapy [18]. Similarly, high p16 expression supposedly correlates with favorable prognosis in esophageal squamous cell carcinoma as well [19, 20], although data are still limited and variable [16, 18–23].

A retrospective cohort study with 136 ESCC patients has showed that p53 overexpression was associated with poor prognosis in these patients and a significantly independent predictor of poor overall survival [16]. However, this prognostic role of p53 overexpression in ESCC remained unclear [16].

Necessary strategies to improve prognosis and survival rates in patients with EC require early diagnosis and treatment, which rely on studying and exploring factors that influence the prognosis of such neoplasia.

This study aimed to evaluate the correlation of HPV infection and the expression of p53 and p16 with clinicopathologic factors, and whether they are ESCC prognostic factors for cancer progression (survival).

## Methods

This was a prospective cohort study. Briefly, the patients of both genders, aged above 18 years, admitted to the Barretos Cancer Hospital, with histopathological confirmation of ESCC, clinical indication for endoscopy and no previous treatment for cancer were included. Medical records were available to obtain clinical and follow-up data.

### Sample collection, HPV detection and characterization

The procedure for conducting the Digestive Endoscopy followed the routine of the Department of Endoscopy at Barretos Cancer Hospital using sedation, flexible video endoscopes (Olympus 180, Japan; Fuginon 4400, Japan) and Single-Use Radial Jaw 4 Biopsy Forceps (Boston Scientific Corporation, Natick, MA). Biological samples were collected from tumors tissues, fixed in 10% buffered formalin and embedded in paraffin. Slides were routinely stained with Hematoxylin-Eosin.

HPV DNA, obtainened by organic extraction [24], was measured in all samples using type-specific PCR bead-based multiplex genotyping (TS-MPG) assays that combine multiplex polymerase chain reaction (PCR) and bead based Luminex technology (Luminex Corp., Austin, TX, USA), as described by Pastrez et al. and da Costa et al. [25, 26].

A primer set targeting the β-globin gene were included as a positive control for the quality of the template DNA and the mix without sample was a negative control. HPV multiplex PCR was performed with QIAGEN Multiplex PCR Kit (Qiagen, Dusseldorf, Germany), according to manufacturer’s instructions, and the details of the reaction can be seen in Pastrez et al. [25] methodology.

For the hybridization assay, the mean fluorescence intensity (MFI) values were obtained when no PCR product was added to the mixture of hybridization was considered as background, for each probe, was performed according to Schmitt et al. (2006) [27]. The cutoff was calculated by adding 5 MFI for 1.1 X the value of median found, and values higher than 20 MFI was considered positive.

### Immunohistochemistry

The immunohistochemistry expression of p16 and p53 proteins were analyzed in automated system (Ventana Benchmark ULTRA, CA, USA) using a primary antibody against p16 (monoclonal mouse anti-human p16INK4A protein, Clone E6H4TM, ready for use, Roche Brazil) and p53 (monoclonal mouse anti-human p53 protein, Clone DO-7, dilution 1:1200, Cell Marque, Rocklin, CA, USA). The scores for analysis oh the proteins and details can be seen in a former study recently published [25].

### Statistical analysis

Survival rates were estimated in months, and survival was defined as the period from the date of diagnosis to the date of death or the date at which information was last obtained from the patient. For the analysis, the event of interest was death related to cancer to specific-cancer survival and the locoregional recurrence, progression or metastasis to progression-free survival. Cases that were alive or dead from other causes were censored to specific-cancer survival and without locoregional recurrence, progression or metastasis to progression-free survival. Such information was obtained through direct consultation to the death certificate or medical records. Multiple confirmatory models were used to check whether HPV, p53 and p16 status were related to prognosis of ESCC. Multivariable Cox proportional hazards regression models was used to estimate hazard ratios (HR) and 95% confidence intervals (CI) with adjustment for sex, clinical stage and treatment. Fisher exact test was used to association analysis. For tabulation and statistical analysis we used IBM® SPSS® Statistics 20.0.1 software for Windows (IBM Corporation, Route 100, Somers NY 10589). The level of statistical significance was set at 0.05 for all analysis.

## Results

During the period between February 2013 and August 2014, 123 patients with ESCC were enrolled in this study. Age ranged from 41 to 92 years (mean = 60.9 years, SD = 10.3 years; median = 61 years). Patients characteristics are described in Table 1; HPV, p53 and p16 status versus patients characteristics are depicted in Table 2.

**Table 1:** Patients’ characteristics

Variable	Category		
		n	%
Sex	Female	23	18.7
	Male	100	81.3
Age at diagnosis	≤ 60 years old	60	48.8
	> 60 years old	63	51.2
Alcohol consumption	≤ 20 years	24	19.5
	>20 years	99	80.5
Tobacco consumption	≤20 years	26	21.1
	>20 years	97	78.9
Clinical stage^a^	I	3	2.6
	II	26	22.8
	III	58	50.9
	IV	27	23.7
Histological grade *	Well differentiated	14	11.6
	Moderately differentiated	73	60.3
	Poorly differentiated	34	28.1
Surgery	No	102	82.9
	Yes	21	17.1
Radiotherapy	No	51	41.5
	Yes	72	58.5
Chemotherapy	No	45	36.6
	Yes	78	63.4
Progression	No	84	68.3
	Yes	39	31.7
Status	Death by cancer	93	75.6
	Alive	30	24.4
HPV^a^	Negative	75	86.2
	Positive	12	13.8
p16^a^	Negative	108	89.3
	Positive	13	10.7
p53^a^	Negative	37	31.4
	Positive	81	68.6

^a^There are missing values

**Table 2:** HPV, p53 and p16 status versus patients’ characteristics

Treatment	HPV^a^		*p*	p16^a^		*p*	p53^a^		*p*
	Negative	Positive		Negative	Positive		Negative	Positive	
Sex									
Female	16 (21.3)	3 (25.0)	0.720	18 (16.7)	5 (38.5)	0.071	6 (16.2)	17 (21.0)	0.624
Male	59 (78.7)	9 (75.0)		90 (83.3)	8 (61.5)		31 (83.8)	64 (79.0)	
Age at diagnosis									
≤ 60 years old	39 (52.0)	7 (58.3)	0.763	51 (47.2)	7 (53.8)	0.772	14 (37.8)	44 (54.3)	0.115
> 60 years old	36 (48.0)	5 (41.7)		57 (52.8)	6 (46.2)		23 (62.2)	37 (45.7)	
Alcohol consumption									
≤ 20 years	19 (25.3)	2 (16.7)	0.722	21 (19.4)	3 (23.1)	0.720	7 (18.9)	17 (21.0)	0.813
> 20 years	56 (74.7)	10 (83.3)		87 (80.6)	10 (76.9)		30 (81.1)	64 (79.0)	
Tobacco consumption									
≤ 20 years	14 (18.7)	5 (41.7)	0.125	25 (23.1)	1 (7.7)	0.295	8 (21.6)	18 (22.2)	0.572
> 20 years	61 (81.3)	7 (58.3)		83 (76.9)	12 (92.3)		29 (78.4)	63 (77.8)	
Clinical stage									
I or II	21 (29.2)	2 (16.7)	0.497	26 (26.0)	3 (23.1)	1.000	5 (14.3)	23 (30.3)	0.099
III or IV	51 (70.8)	10 (83.3)		74 (74.0)	10 (76.9)		30 (85.7)	53 (69.7)	
Histological grade									
Well differentiated	11 (15.1)	0 (0.0)	0.442	13 (12.3)	1 (7.7)	0.912	7 (18.9)	7 (8.9)	0.264
Moderately differentiated	42 (57.5)	9 (75.0)		62 (58.5)	9 (69.2)		19 (51.4)	49 (62.0)	
Poorly differentiated	20 (27.4)	3 (25.0)		31 (29.2)	3 (23.1)		11 (29.7)	23 (29.1)	
Surgery									
No	58 (77.3)	12 (100.0)	0.112	95 (88.0)	7 (53.8)	**0.006**	31 (83.8)	69 (85.2)	1.000
Yes	17 (22.7)	0 (0.0)		13 (12.0)	6 (46.2)		6 (16.2)	12 (14.8)	
Chemoradiotherapy									
No	18 (24.0)	0 (0.0)	0.140	24 (22.2)	2 (15.4)	0.803	7 (18.9)	17 (21.0)	0.871
Chemo or Radio	25 (33.3)	6 (50.0)		38 (35.2)	4 (30.8)		12 (32.4)	29 (35.8)	
Chemo and Radio	32 (42.7)	6 (50.0)		46 (42.6)	7 (53.8)		18 (48.6)	35 (43.2)	
HPV									
Negative	–	–	–	65 (85.5)	9 (90.0)	1.000	24 (88.9)	47 (83.9)	0.743
Positive	–	–		11 (14.5)	1 (10.0)		3 (11.1)	9 (16.1)	
p16^a^									
Negative	65 (87.8)	11 (91.7)	1.000	–	–	–	34 (91.9)	72 (88.9)	0.751
Positive	9 (12.2)	1 (8.3)		–	–		3 (8.1)	9 (11.1)	
p53^a^									
Negative	24 (33.8)	3 (25.0)	0.743	34 (91.9)	72 (88.9)	0.751	–	–	–
Positive	47 (66.2)	9 (75.0)		3 (8.1)	9 (11.1)		–	–	

^a^There are missing values

Entries in boldface are significantly different

**Fig. 1:**
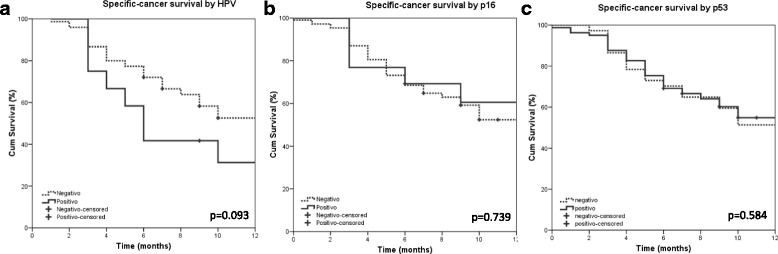
Kaplan Meier curves for specific-cancer survival according HPV, p53 and p16 status

Kaplan-Meier analysis indicated that ESCC male patients had a poor specific-cancer survival (*p* = 0.025) and a shorter progression-free survival (*p* = 0.050); III or IV clinical stage (*p* < 0.019) had a poor specific-cancer survival and a shorter progression-free survival (*p* < 0.001); not submitted to surgery (<0.001) and not submitted to chemoradiotherapy (CTR) (*p* = 0.039) had a poor specific-cancer survival. Those patients with disease progression or metastasis (<0.001) had a poor specific-cancer survival (Table 3). The distribution of cases according to patients’ characteristics and survival rates are shown with more details in Table 3 and the survival curves shown in Fig. 1.

**Table 3:** Survival rates according to clinical and pathological data

Variable	Progression-free survival			Specific survival		
	Total events	One-year	*p*-value	Total events	One-year	*p*-value
Sex						
Female	4	86.7	**0.050**	14	72.3	**0.025**
Male	35	63.3		79	49.8	
Age at diagnosis						
≤ 60 years old	18	73.3	0.553	45	54.9	0.266
> 60 years old	21	63.0		48	53.2	
Alcohol consumption						
≤ 20 years	5	74.6	0.218	16	62.0	0.301
> 20 years	34	66.9		77	52.0	
Tobacco consumption						
≤ 20 years	11	51.3	0.158	19	49.0	0.796
> 20 years	28	73.1		74	55.3	
Clinical stage^a^						
I or II	6	91.2	**0.019**	13	78.6	**<0.001**
III or IV	32	57.9		73	44.2	
Histological grade^a^						
Well differentiated	4	76.2	0.170	10	63.5	0.426
Moderately differentiated	18	72.4		55	48.8	
Poorly differentiated	15	57.4		26	61.4	
Surgery						
No	32	65.8	0.486	84	47.5	**<0.001**
Yes	7	80.0		9	85.4	
Chemoradiotherapy						
No	8	72.4	0.731	24	34.6	**0.039**
Chemo or Radio	15	62.3		36	47.7	
Chemo and Radio	16	72.9		33	69.1	
HPV^a^						
Negative	23	69.3	0.885	56	52.6	0.093
Positive	3	71.4		11	31.3	
p16^a^						
Negative	35	66.9	0.956	84	52.4	0.739
Positive	4	75.2		9	60.6	
p53^a^						
Negative	12	66.3	0.892	27	51.4	0.584
Positive	26	67.0		63	54.9	

^a^There are missing values

Entries in boldface are significantly different

In the multivariate analysis, using a confirmatory model, HPV, p16 and p53 did not show any prediction value related to the progression-free and specific-cancer survival. Results of the multivariable Cox regression analysis are shown in Table 4.

**Table 4:** Risk of cancer progression or death according to HPV, p53 and p16 status

Model	Variable of interest^a^	Progression-free survival			Specific-cancer survival	
		HR	[CI^95%^]		HR	[CI^95%^]
1	HPV	1.042	[0.293: 3.709]		1.901	[0.926: 3.900]
2	p16	1.137	[0.383: 3.378]		1.268	[0.617: 2.604]
3	p53	1.318	[0.646: 2.689]		1.177	[0.726: 1.907]

^a^Model adjusted by sex. Clinical stage and treatment (surgery and chemoradiotherapy). HR: Hazard ratio

## Discussion

Esophageal cancer is an extremely aggressive disease, which is usually diagnosed at an advanced stage, due mainly to the lack of specific initial symptoms. Consequently, EC infiltrates organs and metastasizes straightforwardly, resulting in poor prognosis and 5-year survival of 15–34% [28–30]**.** In cases of advanced disease, it is well established that standard treatment is CRT followed by surgery [31]**,** which leads to downgrade the tumor stage and increase the complete resection rate [2]**.** However, the cure rate and survival of these patients is still low, requiring other methods which may assist in predicting survival and identification of potential responders to a given therapy.

Until now, published data demonstrate that clinic-histopathological factors, molecular biomarkers, and HPV infection are, possibly, predictive variables for neoadjuvant therapy [2, 31]**.** In head and neck cancer, HPV-positive patients have a better response to CRT and a higher survival rate in relation to HPV-negative cancers [32–34]**.** Due to the fact that the esophagus can also be infected with these viruses, a similar association and clinical characteristics [20] are supposed. However, the impact of HPV infection on the prognosis of ESCC is still uncertain [2, 35]**.** In addition, the recent advances in HPV vaccination can believed to improve the reduction of HPV-related tumors in non-gynecological cancers, which is a optimistic scenario to be proved in near future [36].

Previous work of our study group showed a rate of high-risk HPV infection in esophageal tumor samples (13.8%) [25, 26], which led us to investigate whether this event could influence the survival of our patients. However, the current study demonstrated that HPV infection showed no impact on the survival of patients with ESCC and similar results were found in other studies [2, 35, 37, 38].

Hippelainen et al. (1993), e.g., detected HPV in 11% of the esophageal tumors analyzed but the infection was not associated with higher survival rate [38]**.** Dreilich et al. (2006) detected only HPV 16 in their esophageal samples and showed no influence of virus in survival or improvement of therapy response [35]**.** Liu et al. (2010) demonstrated that infection of HPV 16 and p53 protein expression were not correlated with survival during the 5-year follow-up period in ESCC [37]**.** Herbster et al. (2012) found mostly HPV 16 positive in esophageal tumors, but this condition was not associated with overall survival [39]. Recently, Wang et al. (2015) demonstrated that the risk of developing multifocal ESCC was not significantly different between HPV-positive and HPV-negative groups. However, patients with HPV16 infection, specifically, had better response to CRT than those without HPV 16 infection [2]**.**

Different results have also been reported in other studies. Cao et al. (2014) demonstrated that HPV infected patients had better 5-year rates of overall survival and reduction in the risk of death [22]**.** In contrast, Furihata et al. (1993) reported that HPV positive patients have worse survival than those HPV negative with overexpression of p53 in EC patients [40].

In addition to investigating HPV infection in EC, our group has also previously assessed the expression of molecular markers p53 and p16, considered to be essential G1 cell cycle regulatory genes whose loss of function is associated with ESCC carcinogenesis [41], and found that the expression of these proteins was significantly higher in tumor tissues compared to adjacent normal tissue to the tumor and also esophageal tissue from individuals without EC [25]. Based on this interesting result, we decided to evaluate the impact of increased expression of these proteins in EC as regards the survival of these patients. We find, through a multivariate analysis, that p53 and p16 expression showed no predictive value for progression-free and specific-cancer survival. The results found in literature related to the expression of these markers and survival in ESCC are widely variable.

Currently, there are several studies trying to correlate the expression of p53 protein and mutations in the p53 gene with survival of patients carrying EC, and the results are widely variable. Bahnassy et al. (2005) and Huang et al. (2014) found that high p53 expression was associated with a poor survival rate in ESCC patients [42, 43]; and Han et al. (2007) showed that p53 expression was positively correlated with tumor stage and lymph node metastasis [44]. Ye et al. (2012) reported that p53 expression was not associated with the gender or age of the patient, but was associated with tumor differentiation degree and lymph node metastasis [45]. A retrospective cohort study of 136 ESCC patients, conducted to investigate the prognostic role of p53 in patients with ESCC suggested that overexpression of this protein was associated with poor prognosis in these patients, and it’s a significantly independent predictor of poorer overall survival (*p* = 0.04) [16]. Furthermore, significant associations were also found between high expression of p53 and poor prognosis by Shang et al. (2014), Xu et al. (2014) and Chen et al. (2015), suggesting that this protein is an important biomarker candidate for the prognosis of patients with ESCC [3, 14, 23].

Similarly to our results, Chino et al. (2001) showed that p53 expression was not associated with tumor infiltration deepness, lymph node metastasis, or venous and/or lymphatic invasion [46]. Murata et al. (2013) examined the clinical and prognostic features of p53 immunohistochemical expression in 266 ESCC patients and found that the protein expression has no impact on the prognosis of ESCC, according to them, possibly due to their short follow-up time [47]**.** Furthermore, a p53 research group study demonstrated that, for EC, p53 immunohistochemistry does not correlate with response to chemotherapy, curative resection rate, or prognosis, whereas data from p53 mutation analyses are more consistent concerning the association of p53 mutation and poor survival [48]**.** These discrepancies may be related to several factors, including small sample sizes, patient selection bias, failure to take into account other prognostic parameters, differences in laboratory techniques (for example, the use of different monoclonal antibodies to screen for p53 expression) and a shorter time of follow-up [16, 47]. To date, the role of this protein in relation to EC patients’ survival is not fully understood.

Unlike the large number of findings related to p53 overexpression and survival, studies seeking to correlate p16 expression with EC patient survival are scarce, since the vast majority uses this protein as an indirect marker for HPV infection.

Opposite to our findings, Cao et al. (2014) found that p16-positive patients had better 5-year rates of overall survival and progression free survival than p16-negative group [22] and similarly, Kumar et al. (2015) found that the p16 expression in ESCC correlates with a higher rate of pathologic complete remission in patients submitted to neo adjuvant chemotherapy, and could be considered as a predictive marker for response assessment. Furthermore, moderately differentiated histological grade, surgery, chemotherapy and progression or metastasis have shown their prediction value for specific-cancer survival [21]**.** However, no significant correlations were found between the proteins expression and clinical outcomes^[1515]^ **,** corroborating our findings.

## Conclusions

HPV status did not statistically correlated to survival rates, despite the clear tendency of positive HPV cases to be more aggressive than the HPV negative, in opposition to HPV significance in oropharyngeal cancers.

## Abbreviations

CI: Confidence Interval
CTR: Chemoradiotherapy
EC: Esophageal cancer
ESCC: Esophageal squamous cell carcinoma
HPV: Human papillomavirus
PCR: Polymerase chain reaction
